# Look and Outlook on Enzyme-Mediated Macrolide Resistance

**DOI:** 10.3389/fmicb.2018.01942

**Published:** 2018-08-20

**Authors:** Tolou Golkar, Michał Zieliński, Albert M. Berghuis

**Affiliations:** ^1^Department of Biochemistry, McGill University, Montreal, QC, Canada; ^2^Department of Microbiology & Immunology, McGill University, Montreal, QC, Canada

**Keywords:** antibiotic resistance mechanisms, macrolide resistance, macrolide phosphotransferase, erythromycin esterase, ketolides, macrolides, Ere, MPH

## Abstract

Since their discovery in the early 1950s, macrolide antibiotics have been used in both agriculture and medicine. Specifically, macrolides such as erythromycin and azithromycin have found use as substitutes for β-lactam antibiotics in patients with penicillin allergies. Given the extensive use of this class of antibiotics it is no surprise that resistance has spread among pathogenic bacteria. In these bacteria different mechanisms of resistance have been observed. Frequently observed are alterations in the target of macrolides, i.e., the ribosome, as well as upregulation of efflux pumps. However, drug modification is also increasingly observed. Two classes of enzymes have been implicated in macrolide detoxification: macrolide phosphotransferases and macrolide esterases. In this review, we present a comprehensive overview on what is known about macrolide resistance with an emphasis on the macrolide phosphotransferase and esterase enzymes. Furthermore, we explore how this information can assist in addressing resistance to macrolide antibiotics.

## Introduction

After β-lactams and aminoglycosides, macrolides were the third major class of microbial products to be discovered that possess antibiotic properties (Lewis, 2013). The archetypal macrolide, erythromycin, was first isolated in 1949 from the soil dwelling bacterium *Saccharopolyspora erythrea* in a Filipino environmental sample. Within, what is now considered a very short time, this macrolide antibiotic entered clinical practice in 1952. This kick-started the golden-era of macrolide discovery where a plethora of new macrolides were being frequently characterized. Furthermore, it fueled the development of next-generation macrolides using semi-synthetic approaches (Bryskier, 2000).

Initially, macrolides were primarily used for the treatment of upper respiratory tract, skin and soft tissue infections, as dictated by the pharmacological properties of these drugs. As next generation macrolides, improved upon the drug characteristics of these antibiotics, their usage was expanded. Specifically, macrolides now proved effective in the treatment of infections caused by Gram-positive bacteria (e.g., *Streptococcus pneumoniae, Streptococcus pyogenes*, *Staphylococcus aureus*), some Gram-negative (e.g., *Haemophilus influenzae*), as well as atypical pathogens (e.g., *Chlamydia trachomatis –* causative agents of chlamydia*, Treponema pallidum* – causative agents of syphilis, *Mycoplasma pneumoniae*). It has been noted that many of the infections that can be treated by next-generation cephalosporins also are treatable using macrolides (Zhanel et al., 2001). Fortuitously, this provides a much-needed alternative treatment option for patients allergic to penicillins, and has thus increased the clinical application of these drugs (MacLaughlin et al., 2000). It should also be mentioned that the use of macrolides is not strictly restricted to antibiotics. Several macrolides are also in use or in clinical development for modulation of immune response including 12-membered EM-900, 23-membered tacrolimus and 31-membered rapamycin (Gomes et al., 2017).

## Macrolide Antibiotics

### Chemical Structure of Macrolides

**FIGURE 1:**
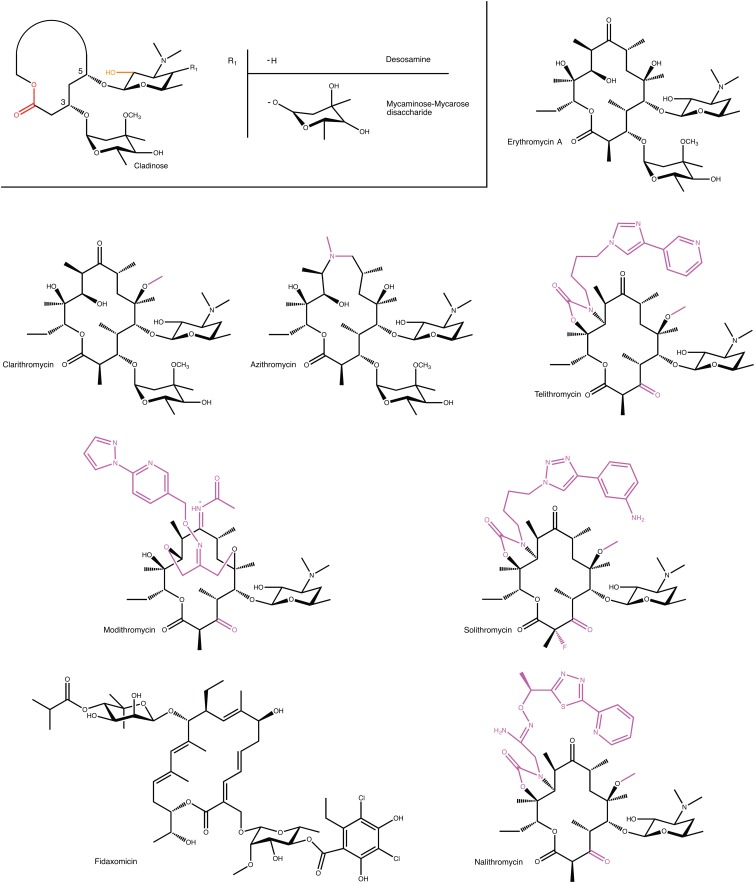
Examples of macrolides used in the clinic and in clinical development. A generic structure is also shown that highlights structural elements present in most macrolides. For semi-synthetic macrolides modifications to the erythromycin scaffold are shown in magenta. MPH- and Ere-mediated modification positions on macrolides are displayed in orange and red, respectively.

Macrolide antibiotics are synthesized by polyketide-synthases present in various *Streptomyces* sp. The archetypal chemical structure for a macrolide antibiotic consists of a 12–18 membered lactone ring to which 1–3 different hexose moieties are directly or indirectly attached (see **Figure 1**). One of the hexose moieties is linked at the C5 position of the macrolactone ring and is either a desosamine or a mycaminose sugar. If it is a mycaminose, a second sugar, mycarose, is linked to this moiety, creating a disaccharide at the C5 position. A cladinose is frequently linked to the C3 position of the ring. Various additional substitutions on the macrolactone ring are observed creating extensive chemical diversity among macrolides (Omura, 2002).

Given the modular nature of macrolide antibiotics, which is a result of these molecules being synthesized by polyketide synthases, it is to be expected that a large number of different macrolides can be found in nature. A conservative estimate is that currently over 2000 different macrolides have been found by various groups (Omura, 2002). However, despite this abundance of molecules, only very few of these have thus far found clinical use. Clearly, not all macrolides possess the required pharmacological properties, to be sufficiently effective in the treatment of bacterial infections. Most notably, one of the persistent roadblocks for the clinical use of macrolide antibiotics has been their inherent susceptibility to acid degradation, which make them suboptimal for oral administration (Hassanzadeh et al., 2007). The desire to obtain improved pharmacokinetic properties has fueled the use of semi-synthetic approaches to create next-generation macrolides.

At present several semi-synthetic macrolides are in clinical use or in the later stages of clinical development (see **Figure 1**). Intriguingly, all of these are derivatives of the first macrolide to be clinically used, erythromycin. In the 1980s, Taisho Pharmaceuticals developed clarithromycin. This derivative of erythromycin is far more stable under acidic conditions, through a mere methylation of C6 hydroxyl group. (Morimoto et al., 1984). Around this time, azithromycin, was also developed by Pliva, as an effective antibiotic with increased acidic stability and improved pharmacokinetic properties (Girard et al., 1987). In the 1990s, under the pressure of rising resistance (see below), radical changes to the erythromycin scaffold were tested. Specifically, removal of erythromycin’s cladinose sugar and oxidation of the remaining secondary alcohol to a keto group resulted in a scaffold that retained antibiotic activity, and that was additionally less susceptible to some forms of macrolide resistance. This sub-class of macrolides have since received their own name: ketolides (Bryskier, 2000).

Recently, two research areas have seen advances that will undoubtedly have a major impact on the development of new macrolides with improved antibiotic properties. The first is the increase in our understanding of how macrolides are synthesized by polyketide-synthases. This has opened the avenue to alter polyketide-synthases through protein engineering approaches, so as to create novel macrolides. Currently, this avenue is still in its infancy, but it shows great promise (Park et al., 2010). The second area is advances in the total synthesis of macrolides. Specifically, Seiple et al. (2016) reported the *de novo* synthesis of several bioactive macrolides from simple starting blocks, providing a feasible method for synthesis of thousands of chemically diverse macrolides. This group was not only able to change the number of atoms in the macrolactone ring, but also add extra moieties to the ring, and modify the sugars. This technology is currently being exploited by Macrolide Pharmaceuticals, a preclinical-stage company that is developing novel antibiotic compounds.

### Mechanism of Macrolide Antibacterial Activity

Through the study of the effects of erythromycin on bacteria it was found early on that macrolides had an impact on protein synthesis (Taubman et al., 1963). Subsequent studies revealed that this was due to binding of the macrolide to the ribosome (Taubman et al., 1966). Around that time, studies of chloramphenicol binding to the 50S ribosome, and interference of this binding by different classes of antibiotics, suggested that macrolides interact with the 50S subunit at a related site (Vazquez, 1966). This binding was also confirmed through studies of binding of erythromycin to ribosomes from antibiotic-sensitive and -resistant *Bacillus subtilis* 168 (Oleinick and Corcoran, 1969), through the fragment reaction studies (Celma et al., 1970) and through dimethyl sulfate and kethoxal probing (Moazed and Noller, 1987). Furthermore, this binding was shown genetically through two chloramphenicol-erythromycin resistance mutations on *Escherichia coli* 23 rRNA (Ettayebi et al., 1985). However, it took some time before the exact location and mechanism of ribosome binding and inhibition was determined through X-ray crystal structures of 50S and 30S ribosomal subunits and the intact 70S ribosome (Ban et al., 2000; Wimberly et al., 2000; Schlünzen et al., 2001, 2003; Tu et al., 2005).

Macrolides bind in the peptide exit tunnel of the large ribosomal subunit, immediately adjacent to the peptidyl transferase center. They block the lumen of the tunnel preventing an elongating polypeptide chain to pass through it, causing either a bacteriostatic effect or a bactericidal result, depending on the macrolide (Svetlov et al., 2017). It is noteworthy that this exact site in the bacterial ribosome is not only exploited by macrolides to exert an antibacterial effect, as also class B streptogramins and lincosamides bind in this location (Tu et al., 2005; Matzov et al., 2017). As discussed below, this has implications since certain mechanisms of resistance to these antibiotics also confer resistance to macrolides.

**FIGURE 2:**
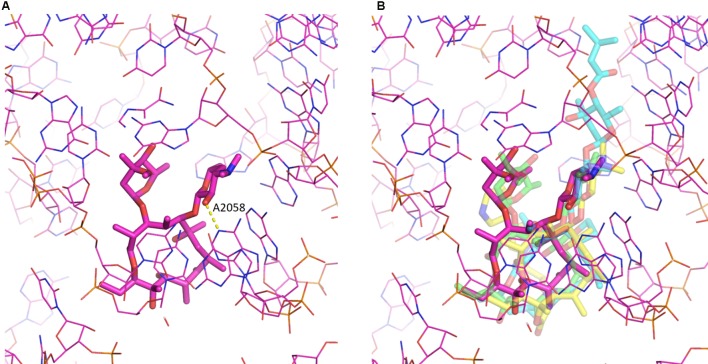
Macrolide binding to the 23S rRNA. **(A)** The binding of erythromycin to the ribosome is shown. Highlighted also is A2058 and the hydrogen bond it makes with the 2′ hydroxyl of the desosamine moiety. **(B)** Additional macrolides are shown illustrating the similarity in their binding modes. Depicted are erythromycin (purple; pdb code: 4V7U), azithromycin (green; pdb code: 1M1K), carbomycin A (cyan; pdb code: 1K8A) and the ketolide telithromycin (yellow; pdb code: 4V7S). The figure was prepared using PyMOL (Schrödinger, 2017).

Despite the chemical diversity of macrolides, there is extensive similarity in how they bind to the ribosome (see **Figure 2**). First of all, the lactone rings, which possess a hydrophobic and a hydrophilic face, invariably bind to the ribosome with their hydrophobic face. The desosamine/mycaminose moiety at the C5 position makes specific hydrogen bond interactions with the nucleotide residues A2058 and A2059 (*E. coli* numbering). Furthermore, for those macrolides that possess a sugar at the C3 position, this cladinose group makes specific interactions with the base of nucleotide 2505, though this only contributes incrementally to the affinity of the macrolide for the 50S subunit (Hansen et al., 2002).

Intriguingly, for some 16-membered lactone ring macrolides, that also possess an acetaldehyde group at the C6 position, such as spiramycin, a covalent bond has been crystallographically observed between the macrolide’s aldehyde group and the primary amine group at the N6 position of A2062, creating a carbinolamine linkage (Hansen et al., 2002). For this, the base of nucleotide 2062 of the 23S rRNA must reorient by almost 90 degrees so that it protrudes into the lumen of the tunnel. As the carbinolamine linkage is reversible, this observation does not imply irreversible binding of these macrolides to the ribosome.

For the ketolides sub-class, the absence of the specific interactions afforded by the cladinose group are compensated by interactions created by the cyclic carbamate moiety present in these antibiotics. The ketolides (because of the carbamate moiety and the quinolyl allyl group) have been reported to interact not only to the domain V of 23S rRNA but also with domain II (helix 35) and possibly domain IV (Hansen et al., 1999; Xiong et al., 1999; Zhanel et al., 2001; Berisio et al., 2003; Schlünzen et al., 2003).

It is appropriate to mention here that while macrolides are known to interfere with protein synthesis through binding to the bacterial ribosome, this is not universally true. Noteworthy, the 18-membered ring macrolide, fidaxomicin, inhibits RNA polymerase (Artsimovitch et al., 2012).

## Clinical Resistance to Macrolide Antibiotics

Bacterial resistance to erythromycin was initially reported in *Staphylococci* in 1956, only a few years after its introduction into clinical practice (MacCabe and Gould, 1956). The first erythromycin-resistant strains of *Streptococci* were reported in the United Kingdom in 1959 and in North America in 1967 (Lowbury and Hurst, 1959; Dixon, 1968). Since that time, resistance has been detected in a large number of bacteria including *Staphylococcus* spp., *Streptococcus* spp., *Bacteroides* spp., *Enterococcus* spp., *Clostridium* spp., *Bacillus* spp., *Lactobacillus* spp., *M. pneumoniae*, *Campylobacter* spp., *Corynebacterium diphtheriae*, *Propionibacterium* and members of the Enterobacteriaceae (Leclercq and Courvalin, 1991; Weisblum, 1995; Zhanel et al., 2001).

The extend of macrolide resistance has becoming alarming depending on the bacterial pathogen and the location. For example, erythromycin-resistance *Campylobacter jejuni* rates have reached 22% in New Delhi, India (Ghosh et al., 2013). Also, clarithromycin-resistant *Helicobacter pylori* has been on the rise in many countries over the past decade, with rates as high as ∼30% in Japan and Italy, 40% in Turkey and 50% in China (Thung et al., 2016). In another study, the rate of macrolide resistance *S. pneumoniae* among outpatients of county hospitals in China was reported to be 89–96%. In the same study, the rate of macrolide resistance MRSA (methicillin-resistant *S. aureus*) was found up to 82% and for MSSA (methicillin-susceptible *S. aureus*) up to 63% (Xiao et al., 2015). Finally, the incidence of macrolide-resistant *M. pneumoniae* in Japan can go as high as 90% and in Zhejiang province of China to 100% (Pereyre et al., 2016).

As is discussed below, one of the mechanisms of resistance to macrolides is by target modification, i.e., alterations in the bacterial ribosome that compromise binding of the antibiotic. However, as previously mentioned, macrolides exploit the same pocket in the ribosome as several other antibiotics, specifically lincosamides and B streptogramins. This implies that the target modification observed in macrolide resistant bacteria may also confer resistance to lincosamides and B streptogramins. Indeed, this has been observed and the associated phenotype is now referred to as MLS_B_ (Weisblum, 1995; Leclercq, 2002). Unfortunately, this also implies that certain forms of macrolides resistance are a far greater clinical and societal problem as they effectively negate usage of three different classes of antibiotics, substantially reducing the available armament of antibiotics for treating bacterial infections.

## Mechanisms of Resistance to Macrolide Antibiotics

As with other antibiotics, resistance to macrolide antibiotics is not confined to one single mechanism, but several mechanisms of resistance have been observed. Specifically, mechanisms to: (i) decrease the intracellular concentration of macrolides, (ii) alter the target (ribosome), (iii) protect the target (ribosome), and (iv) chemically modify the antibiotic are observed in clinical isolates.

### Decreased Intracellular Concentration

One way in which bacteria are able to evade the action of macrolides is to reduce the intracellular concentration through the use of efflux pumps. Several different families of pumps have been discovered including major facilitators superfamily (MFS), ATP-binding cassette (ABC) superfamily, multidrug and toxic compound extrusion (MATE) family, resistance-nodulation-division (RND) superfamily and small multidrug resistance (SMR) family (Gomes et al., 2017). These efflux pumps can be encoded on a chromosome or plasmids, and frequently provide resistance to multiple classes of antibiotics. Furthermore, they can often be upregulated in the presence of antibiotics (Ambrose et al., 2005).

Of particular relevance to macrolides are the Mef and Msr subfamilies of efflux pumps, which are encoded on plasmids and which are members of the MSF and ABC families, respectively. Since Mef proteins are members of the MSF family they do not use ATP as an energy source to pump the antibiotics to the exterior of the cell, instead they utilize secondary active transport, where the energy of ATP is not used directly to transport macrolides across the membrane. This subfamily of proteins is one of the important determinants of the macrolide resistance, with Mef(A) and Mef(E) being the most commonly found. Msr subfamily of proteins are members of ABC family that use ATP as an energy source for active transport. Both Mef and Msr subfamily of proteins are capable of using 14- and 15-membered macrolides as substrates, including the ketolide telithromycin. We refer the reader to reviews for further information on macrolide pumps (Li and Nikaido, 2009; Gomes et al., 2017).

### Ribosome Modification

Three types of macrolide resistance conferring modifications to the ribosome have been observed in bacteria. Most prominently is methylation of the 23S rRNA by the members of the Erm family of methyltransferases. These enzymes catalyze the methylation of the N6 position of nucleotide A2058 in the 23rRNA. This nucleotide makes specific interactions with the saccharide moiety located at the C5 position of the macrolactone ring, and methylation interferes with productive hydrogen bond formation. Mono-methylation of this nucleotide confers low-to-moderate resistance to macrolides, whereas di-methylation confers high resistance. It is important to note that di-methylation by Erm methyltransferases additionally confers high resistance to all MLS_B_ antibiotics as well as the ketolide telithromycin, exacerbating antibiotic resistance (Poehlsgaard and Douthwaite, 2005; Roberts, 2008).

Besides methylation of the rRNA, mutations in the rRNA can also confer resistance. Mutation of A2058 will alter the ribosomal target site and prevent binding of macrolides, as well as lincosamides and group B streptogramins (Franceschi et al., 2004; Lambert, 2005; Tu et al., 2005). Furthermore, it has been shown that mutations of A2059 will confer macrolide and lincosamide resistance (Vester and Douthwaite, 2001; Leclercq, 2002). Numerous other mutations have been reported in both domains II and V that confer resistance to various macrolides, and this list is continuously expanding (Vester and Douthwaite, 2001; Hansen et al., 2002).

Mutations in some of the ribosomal proteins are also capable of conferring resistance. Specifically, alterations have been identified in the L4 and L22 ribosomal proteins. These alterations are single amino acid changes or could also consist of insertion/deletion of one or more amino acids to these proteins. These mutations have been documented in many clinical isolates, including *S. pneumoniae*, *H. influenzae*, and *E. coli* (Roberts, 2008). Mutations in L4 and L22 have been proposed to confer resistance through changing the shape of the peptide exit tunnel and distortion of the macrolide-binding site, which results in altered binding kinetics for macrolides (Gabashvili et al., 2001; Moore and Sauer, 2008; Lovmar et al., 2009; Wekselman et al., 2017).

### Ribosome Protection

Recently a new mechanism of resistance has been described for macrolides, mediated by members of the ABC-F subfamily of ATP-binding cassette proteins, such as MsrE (Sharkey et al., 2016; Su et al., 2018). Electron microscopy and biochemical studies for MsrE show that this protein can bind to a stalled ribosome in which a peptidyl-tRNA is in the P-site. The ATP bound form of MsrE can than insert a needle-like domain that reaches the peptidyl transferase center and the adjacent peptide exit tunnel, i.e., the location where macrolides bind, where it pushes the antibiotic out of its binding site (Su et al., 2018). Note that since streptogramins and lincosamides also bind in this region of the ribosome, MsrE and/or homologs of this protein can confer resistance to other MLS_B_ antibiotics. Although this is a novel mechanism of resistance to macrolides, it is reminiscent of what has previously been described for tetracyclines (Nguyen et al., 2014; Arenz et al., 2015).

### Drug Modification

A third mechanism of resistance to macrolides is the enzymatically catalyzed modification of these antibiotics. As a consequence of the alteration facilitated by specific enzymes, the modified macrolides are no longer capable to bind effectively to the 50S ribosome, and are thus unable to exert an antibiotic effect. Thus far two classes of enzymes have been identified in bacteria that confer resistance to macrolide antibiotics: macrolide phosphotransferases (MPHs) and Macrolide Esterases (Eres). Below these two classes of enzymes are discussed in greater detail.

It is worth noting that a third class of enzymes has been identified that modify macrolides, i.e., macrolide glycosyltransferases (Cundliffe, 1992; Quirós et al., 2000; Bolam et al., 2007). However, these enzymes are not involved in conferring antibiotic resistance as they are only present in macrolide producing bacteria where they provide “host cell antibiotic immunity” (Fyfe et al., 2016). However, it is possible that in future this self-protection mechanism could be co-opted by other bacteria and transformed into a *bona-fide* antibiotic resistance mechanism.

## Macrolide Phosphotransferases

**FIGURE 3:**
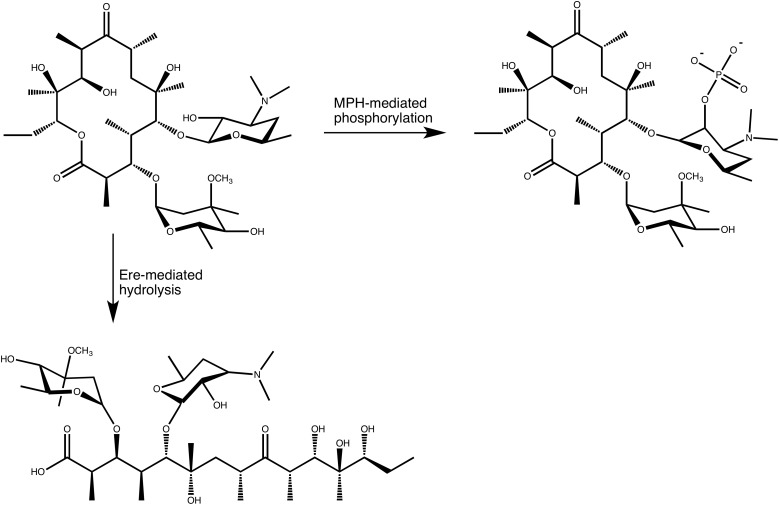
Enzymatic modifications of erythromycin A catalyzed by MPH(2′)s and Eres.

In the search for novel mechanisms of resistance to macrolides, O’Hara, Kanda and Kono examined the ability of erythromycin-resistant clinical strains to detoxify macrolides, in the late 1980s. This search initially revealed that bacterial lysate from a clinical *E. coli* strains was able to phosphorylate oleandomycin, in 1988 (O’Hara et al., 1988). Subsequent work resulted in the purification and characterization of an enzyme that phosphorylated the hydroxyl group located at the 2′ position of the C5 linked desosamine moiety of erythromycin and oleandomycin (see **Figure 3**). This enzyme was accordingly named macrolide 2′-phosphotransferase (O’Hara et al., 1989). Following this discovery several more enzymes have been found that show a similar activity. These MPHs all mediate the transfer of the γ-phosphate group from GTP onto the macrolide substrates and doing so confer resistance to a group of bacteria ranging from Gram-negative (*E. coli, Pseudomonas, Pasteurella, Klebsiella, Serratia, Shigella*) to Gram-positive (*Staphylococcus*) (Matsuoka et al., 1998, 2003; Nguyen et al., 2009; Ferjani et al., 2012; Mendes et al., 2017).

### Macrolide Phosphotransferase Family Members

At least 15 gene subtypes of MPHs have been reported, which are designated *mph*(A) to (O) (O’Hara et al., 1989; Kono et al., 1992; Kim et al., 1996; Matsuoka et al., 1998, 2003; Roberts et al., 1999; Schlüter et al., 2007; Pawlowski et al., 2016, 2018a). Here, we name their products MPH(2′)-I to MPH(2′)-XV, respectively, with the assumption that all these MPHs phosphorylate the hydroxyl on the C5 linked desosamine or mycaminose moiety, which is present in all macrolides and ketolides that bind to the 23S rRNA where it forms a critical interaction with A2058 (see **Figure 2**). However, this is strictly only confirmed for MPH(2′)-I, II, VIII, IX, and XI. Among these fifteen gene subtype of MPHs, *mph*(A), (B), and (C), which are encoded on mobile genetic elements, are found in clinical isolates of *E. coli*, *Salmonella* sp., *Klebsiella* sp., and *S. aureus.* Six more MPHs are encode on mobile genetic elements, but are thus far only found in non-pathogenic bacteria, e.g., MPH(2′)-XIV has been observed in *Exiguobacterium* and *Brachybacterium*. However, this could readily change. The remaining six mph genes are chromosomally encoded in non-pathogenic bacteria, such as MPH(2′)-VIII which is present in *Brachybacterium faecium* and MPH(2′)-XI which is present in *B. subtilis* 168.

**FIGURE 4:**
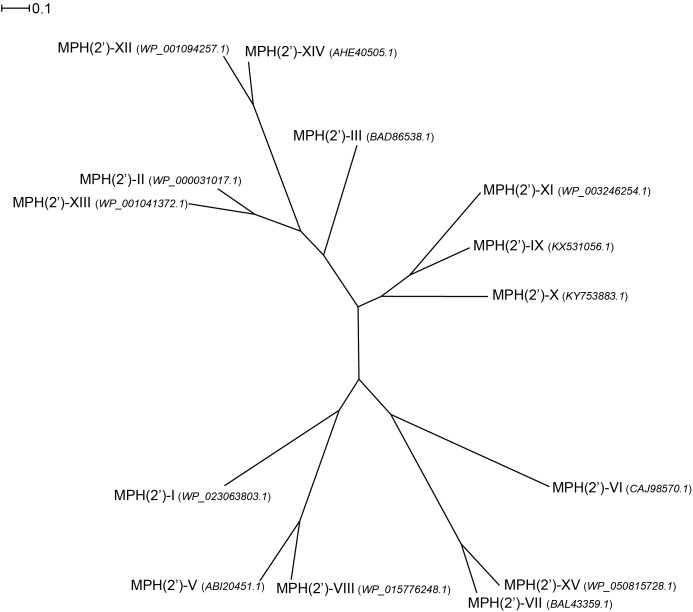
Radial phylogram of MPH family of proteins. NCBI accession codes for the sequences are provided in brackets. Distance scale represents the number of differences between sequences (e.g., 0.1 means 10% differences between two sequences). Phylogenetic relationships were calculated using phylogeny.fr (Dereeper et al., 2008) and displayed using Dendroscope 3 (Huson and Scornavacca, 2012).

Examination of the sequence diversity among 14 MPHs enzymes indicates that the various members can display extensive differences [only a partial sequence is available for the *mph*(D) gene]. For example, MPH(2′)-I and II only share 36% identity. Though several sequences cluster together, e.g., MPH(2′)-IX, X, and XI, with pairwise %identity of 46–54 (see **Figure 4**). Perhaps somewhat surprisingly, there is no real relationship between MPHs that cluster with whether they are chromosomally encoded or on mobile elements, or whether they are in pathogenic bacteria or in environmental isolates.

Macrolide phosphotransferases can confer resistance to a wide range of macrolide substrates, but this topic has not yet been thoroughly investigated, and much remains unknown about their substrate specificity profile. Comparing substrate specificity of the clinically relevant MPH(2′)-I and II reveals that MPH(2′)-I can only efficiently inactivate 14- and 15-membered lactone macrolides, whereas MPH(2′)-II can additionally inactivate 16-membered lactone macrolides and the ketolide, telithromycin (Kono et al., 1992; Fong et al., 2017). A similar observation has been made for MPH(2′)-XII and XIII, with MPH(2′)-XII mirroring the substrate profile of MPH(2′)-I and MPH(2′)-XIII mirroring the substrate profile of MPH(2′)-II (Wang et al., 2015). Also, MPH(2′)-III has been shown to have the same broad substrate specificity as MPH(2′)-II (Chesneau et al., 2007). MPH(2′)-IX from the environmental bacterium *Paenibacillus* sp. LC231 and MPH(2′)-XI from *B. subtilis* 168 are unable to confer resistance to macrolides with a C3 cladinose in cell-based assays. Although, biochemical analysis of drug modification for both enzymes showed that they can use C3 cladinose containing macrolides as substrates but cannot inactivate 14-membered and 15-membered lactone macrolide as efficiently as macrolides without this moiety (Pawlowski et al., 2016, 2018a). Intriguingly, MPH(2′)-X, which is a closer homolog to MPH(2′)-IX than MPH(2′)-XI, is able to effectively provide resistance to several cladinose containing macrolides (Pawlowski et al., 2018b). This observation underscores that sequence similarity among MPHs provides no indication to what the substrate profile for these enzymes might be.

### Structural Insights Into Macrolide Phosphotransferase Mediated Resistance

**FIGURE 5:**
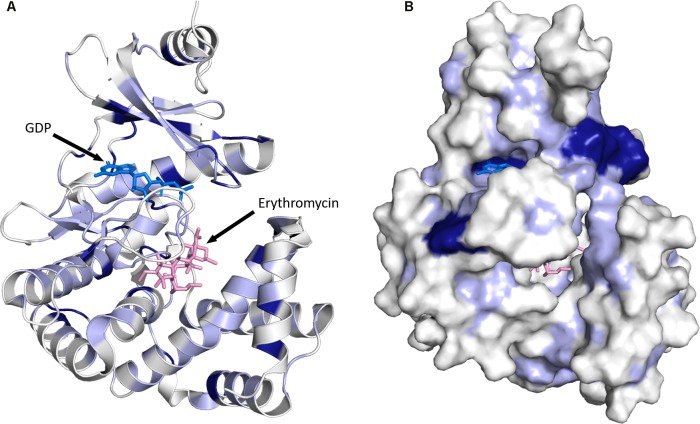
Three-dimensional structure for MPHs. **(A)** MPH(2′)-I in complex with GDP and erythromycin is shown. The color coding used illustrates sequence conservation within the 14 MPHs enzymes, with dark blue indicating completely conserved residues, light blue residues that are conserved among more than seven members, and white residues that are not conserved. **(B)** The enzyme is shown in surface representation. The figure was prepared using PyMOL (Schrödinger, 2017).

Fong et al. (2017) have recently reported the first three-dimensional structures for MPH enzymes. Specifically, MPH(2′)-I and MPH(2′)-II were determined, in their apo state, in complex with GTP analogs and in complex with several macrolides (see **Figure 5**). These structures confirm what sequence comparisons had suggested that MPHs are members of a large superfamily that also includes eukaryotic protein kinases (ePKs) and aminoglycoside phosphotransferases (APHs). The archetypal structure for the members of this superfamily is composed of an N-terminal lobe that contains a five-stranded β-sheet and C-terminal lobe that contains several α-helices. In between these two lobes is the binding site for a tri-phosphate nucleotide that is used as the phosphoryl donor. The C-terminal lobe contains the substrate binding site, but the specific local architecture for this section can differ significantly between various members of the superfamily. For the two MPH enzymes, the architecture of their N-terminal lobe is similar to that seen for the N-terminal lobes of Ser/Thr and Tyr protein kinases, and APHs (Hon et al., 1997). The C-terminal lobe is largely identical to what is seen for a sub-family of APHs, the APH(2^′′^) group. with which they share approximately 17% sequence identity (Shi and Berghuis, 2012). On the other hand, MPHs deviate from archetypical ePKs and APHs in the region between the N- and C-terminal lobes. In ePKs and APHs, the lobes are connected by a loop 5–12 residues in size, while in MPH (2′)-I and MPH(2′)-II the linker region is significantly larger, spanning approximately 25 residues (Fong et al., 2017).

As stated above, the sequence conservation in MPHs is not extensive (see **Figure 4**). However, as the structures of MPH(2′)-I and MPH(2′)-II reveal, this does not impact the fold of these enzymes, as their structures are highly similar (Fong et al., 2017). To further examine the sequence conservation, we mapped the extent of conservation onto the three-dimensional structure (see **Figure 5**). Not unsurprisingly, there are a very limited number of conserved residues among the 14 MPHs, and these include residues required for catalysis, e.g., coordination of the GTP associated Mg^2+^ ions. Intriguingly, the aspartate responsible for abstraction of the proton of the macrolide 2′ hydroxyl group, which is absolutely conserved in ePKs and APHs is apparently a glutamate in MPH(2′)-VIII and MPH(2′)-XV. Furthermore, there is conservation in the nucleotide binding pocket, rationalizing why all MPHs studied use GTP as the phosphoryl donor.

Examination of residue conservation in the macrolide binding area of MPHs reveals that this is not at all conserved. However, delving deeper into this, the chemical character of the macrolide binding pocket is similar in MPHs: generally hydrophobic with a region of negative charge around the conserved proton abstracting catalytic base (Fong et al., 2017). Structural studies of MPH(2′)-I and MPH(2^′′^)-II showed that the relatively non-specific hydrophobic nature of the binding site and the fact that many of the interactions between the macrolides and the enzymes involve the lactone ring would facilitate the accommodation of a range of macrolide substrates.

The large contribution of non-specific hydrophobic interactions to the binding of macrolides to MPHs complicates the rationalization of these enzymes’ substrate specificity, based on the three-dimensional structure. For example, while there is now ample structural data for MPH(2′)-I and MPH(2′)-II and their interactions with 14-, 15- and for MPH(2′)-II 16-membered macrolactone rings, a structural reason for the inability for MPH(2′)-I to phosphorylate 16-membered macrolides is not yet forthcoming (Fong et al., 2017). Recently, despite having structural data for MPHs, Wright and colleagues had to resort to using ancestral sequence reconstruction thus building an evolutionary path for MPH functional divergence and subsequent site-saturation combinatorial mutagenesis, to identify residues that dictate the preference of MPH(2′)-IX for macrolides lacking the cladinose moiety (Pawlowski et al., 2018a). Intriguingly, the residues identified for impacting cladinose specificity were non-obvious as they were not in close proximity of the cladionse binding pocket. This further emphasizes the complexity in linking sequence to MPH substrate specificity.

## Macrolide Esterases

In the mid 1980s Courvalin and co-workers identified a plasmid in a clinical *E. coli* strain that conferred high level resistance to erythromycin, but not lincosamides or group B streptogramins, implying that the resistance mechanism was not caused by the at that time known Erm methyltransferases that methylate A2058 of the 23S rRNA (Andremont et al., 1986). Subsequent characterization of the enzyme encoded on the plasmid revealed that it exploits a feature present in all macrolides. In the biosynthesis of macrolides by polyketide synthases the macrolide aglycon is converted to a cyclic lactone, forming an ester bond. The enzyme identified by Courvalin and his team hydrolyzes this ester bond, thus linearizing the macrolide again, which is then no longer able to bind to its ribosomal binding site (Barthelemy et al., 1984) (see **Figure 3**). Actually, the exact product of the reaction catalyzed by the esterase has not been thoroughly identified. The current proposal is that the hydrolysed macrolide product is naturally very unstable and undergoes spontaneous rearrangement and dehydration. A detailed mechanism for this has been proposed (Barthelemy et al., 1984). Nonetheless, based on the presumed activity, the enzyme was therefore identified as an erythromycin esterase (Ere). Shortly after the discovery of the first erythromycin esterase, Courvalin and co-workers identified a second enzyme with a very similar activity, and additional members of this family have since been identified.

### Erythromycin Esterase Family Members

The first two members of the erythromycin esterase family, discovered by Courvalin and colleagues from clinical *E. coli* strains are known as EreA (Ounissi and Courvalin, 1985) and EreB (Arthur et al., 1986). Since then, three more erythromycin esterases have been discovered: EreA2 in multidrug-resistant *Vibrio cholerae* (Thungapathra et al., 2002), EreC in multidrug-resistant *Klebsiella penumoniae* (Yong et al., 2009) and EreD in the duck pathogen *Riemerella anatipestifer* (Xing et al., 2015). Except for EreD, which is chromosomally encoded, all other Ere enzymes are encoded on mobile genetic elements, and thus are found in numerous different bacterial species, including environmental and clinical isolates. EreA enzyme is mostly found in environmental isolates, however, it has also been detected in *E. coli* and *S. aureus*. EreA2 is the vastly more clinically relevant cousin which has been detected in a multitude of important pathogens such as: *Pseudomonas* spp. (Kim et al., 2002; Kim and Cerniglia, 2005), *Salmonella indiana* (Zhao et al., 2017), *Klebsiella pneumoniae* (Abbassi et al., 2008), *E. coli* (Chang et al., 2000; Ahmed and Shimamot, 2011), non-typhoidal *Salmonella enterica* (Krauland et al., 2010), *Salmonella* spp. (Murphy et al., 2007), and *Vibrio cholera* (Thungapathra et al., 2002). EreB can be found in a range of pathogens including: *E. coli* (Arthur et al., 1986; Nakamura et al., 2000), *MRSA* (Schmitz et al., 2000b), MSSA (Schmitz et al., 2000a), *Staphylococcus saprophyticus* (Fernandez-Fuentes et al., 2014), *Klebsiella oxytoca* (Fuentes et al., 2014), and *Salmonella* spp.(Fuentes et al., 2014). The remaining two enzymes EreC and EreD, due to being a much newer addition to the family of Eres have stayed under the radar of macrolide-research community with only their discovery being published.

**FIGURE 6:**
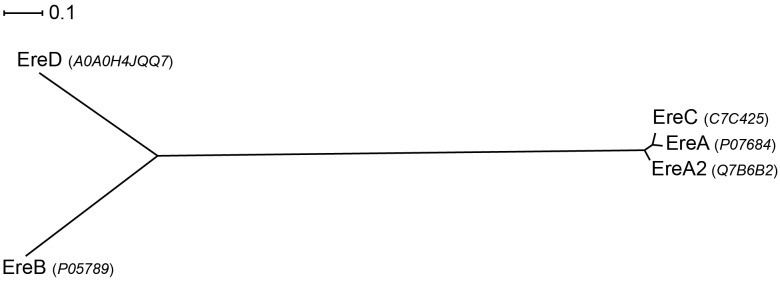
Radial phylogram of Ere family of proteins. UniProt accession codes for the sequences are provided in brackets. Distance scale represents the number of differences between sequences (e.g., 0.1 means 10% differences between two sequences). Phylogenetic relationships were calculated using phylogeny.fr (Dereeper et al., 2008) and displayed using Dendroscope 3 (Huson and Scornavacca, 2012).

Sequence analyses of the five Ere family members reveals that there is extensive diversity in this small group. For example, EreB and EreC display the highest sequence divergence, with 44.8 and 23.0% sequence similarity and identity, respectively. In contrast, EreA, EreA2, and EreC enzymes share extensive sequence similarity with the %identity ranging between 90.0 and 92.6% (see **Figure 6**).

Presently, very limited information is available on the substrate specificities of Ere enzymes. One of the most in-depth studies on kinetics and substrate specificities of Eres focused solely on EreA and EreB and examined just five macrolides that also included one ketolide (Morar et al., 2012). The result showed that both enzymes are capable of cleaving 14-membered macrolides, and that EreB is able to also cleave 15-membered macrolides. However, neither enzyme is able to digest telithromycin, the sole ketolide tested. Surprisingly, despite the clinical relevance of EreA2 and EreC, no information is available of substrate specificities of these two enzymes. For now it is assumed that due to high sequence similarity with EreA, they likely have a very similar substrate spectrum, being only able to degrade 14-membered macrolides (see **Figure 6**).

### Structural Insights Into Erythromycin Esterase Mediated Resistance

As of yet no three-dimensional structure has been determined of an erythromycin esterase, however, a search for homologous proteins with a known structure identified BcR135 and BcR136 as the closest homologs (PDB codes 3B55 and 2QGM, respectively). These two proteins are found in *Bacillus cereus* and are hypothesized to be involved in succinoglycan biosynthesis. The BcR135 and BcR136 sequences are 58.6% similar, and as is to be expected, they possess the exact same fold (Morar et al., 2012). The extent of sequence similarity of BcR135 and BcR136 vs. Eres is considerable, and ranges between 30.1 and 38.7% sequence similarity. Furthermore, similar to Eres they are both capable of cleaving the broad esterase substrate *p*-nitrophenyl butyrate (*p*-NPB). However, BcR136 has been proven not to possess macrolide esterase activity, and given the similarity between the two proteins, this also is likely true for BcR135. Intriguingly, the structure of BcR135 and BcR136 display a novel fold for esterase enzymes.

**FIGURE 7:**
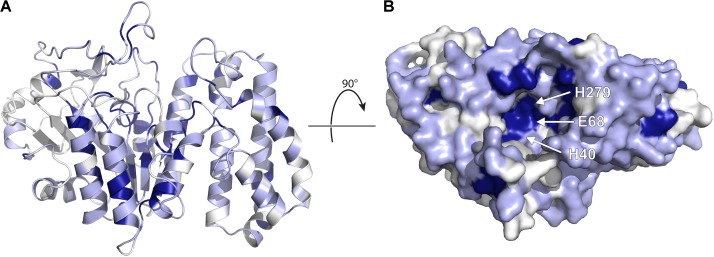
Homology model of EreA based on the structure of BcR135. **(A)** The model is depicted, highlighting secondary structure elements. The colors used illustrate sequence conservation within the five Ere enzymes, with dark blue indicating completely conserved residues, light blue residues that are conserved among only 3–4 members, and white residues that are not conserved. **(B)** The enzyme is rotated and shown in surface representation, highlighting the putative active site and the location of putative catalytic residues. The figure was prepared using PyMOL (Schrödinger, 2017).

Given the extent of sequence similarity between BcR135 and BcR136 and the Eres enzymes, their structure can be used to generate a moderately accurate homology model of the resistance enzymes. We build a model of EreA based on BcR135, as it has been proposed that EreA is a metal-dependent esterase (Morar et al., 2012), and BcR135 has a Ca^2+^ present in its structure (note: EreB has not been reported to require a metal ion for catalysis) (PDB: 3B55). Subsequently, we used this EreA homology model to examine the sequence conservation among the five Ere enzymes (see **Figure 7**).

As shown in **Figure 7**, several completely conserved residues are located at the bottom of a groove, i.e., H40, E68, and H279. Note that these residues are also conserved in BcR135 and BcR136. It is tempting to speculate that this identifies the active site of the enzyme. This suggestion is further bolstered by the observation that the Ca^2+^ ion in BcR135 is coordinated to two of these residues. In fact, Morar et al. (2012) have mutated these specific residues in EreB and shown that these mutations impact the catalytic activity of the enzyme. The authors speculate the role of H40 to be of a catalytic base that activates a water molecule which then becomes a nucleophile that will attack the ester bond of the macrolide. How this proposed mechanism could apply to the presumed metal dependent EreA enzyme is unclear. It is likely that EreA does not in fact require a metal ion for catalysis and that the calcium ion present in BcR135 is an artifact of the structure determination and does not reveal critical information on mechanism of catalysis.

The homology model of EreA also reveals that the walls of the groove, above the presumable active site, are somewhat less conserved. This could explain some of the reported differences in substrate specificity between EreA and EreB. However, given the very limited data available for the substrate spectrum of different Eres, combined with the complete absence of information on how a macrolide might actually bind to an Ere, further discussion on the structural basis of Ere substrate specificity is not feasible, and will need to wait until the three-dimensional structure of an Ere-macrolide complex has been determined.

## Counteracting Macrolide Resistance

Three-dimensional structural information on how macrolides are rendered useless by various bacterial resistance mechanisms can be exploited for the development of therapies that are more resilient against such resistance. If additional detailed information is available for how macrolides bind to the ribosome, this can be also incorporated. Specifically, two distinct avenues are available for the rational development of improved therapies. First, information on features of the macrolide that are recognized by resistance mechanisms and contrasting this with how these drugs bind to ribosome can inform the design of next-generation macrolides, i.e., variants that are unable to bind to resistance proteins but that retain affinity for the ribosome. The second avenue for combating macrolide resistance is to develop inhibitors to the resistance proteins which can then be used as adjuvants to restore the activity of existing antibiotics. The validity of this strategy is illustrated by a familiar β-lactam antibiotic therapy where amoxicillin or ampicillin is co-administered with sulbactam or tazobactam, which inhibit some of the commonly encountered β-lactam resistance enzymes (Drawz and Bonomo, 2010). Below these two avenues are further explored.

### Avenues for Next-Generation Macrolide Antibiotic Development

When considering the design of a next-generation macrolide, it would be desirable if this design could address as many forms of clinically observed resistance as feasible. For macrolides, the challenge is to address: efflux pump action, di-methylation/mutation of the ribosome, and the action of MPHs and Eres. Unfortunately, efflux pumps are well-known for having a very broad range of action toward xenobiotics, leaving ribosome alteration and antibiotic modification, as the resistance mechanisms that can potentially be addressed by improved design. Fortunately, three-dimensional structural information is available to assist in this design effort.

Ribosome alterations are largely centered on A2058 of the 23S rRNA, which makes a specific hydrogen-bond interaction with the C5-linked desosamine/mycaminose moiety of macrolides. This suggests that alterations in that group could be an avenue for exploration. However, the effectiveness of abolishing the hydrogen-bond between A2058 N6 and 2′ hydroxyl of macrolides for precipitating resistance, suggests that merely removing this hydroxyl group on the macrolide will not be sufficient and compensatory interactions have to be engineered to maintain effective and specific binding affinity of the macrolide with the ribosome. Interestingly, the MPHs also target this same hydroxyl group for phosphorylation, and as such alterations in this part of the macrolide might simultaneously circumvent resistance by these enzymes. Alternatively, MPH mediated resistance could be addressed by interfering with the unique manner in which macrolides bind to these enzymes. Although macrolides bind in a similar way to the ribosome as to MPHs, the MPH-macrolide binding seems to be much more fitted. This becomes especially visible with 16-membered macrolides, where a section of C-9 to C-14 extends to the lumen of the exit tunnel whereas this particular section forms relatively close interaction with MPHs. As proposed by Fong et al. (2017) this feature is a potential avenue by which next generation macrolides could be altered to prevent interaction with MPH while at the same time retaining the ribosome binding.

As mentioned previously Ere enzymes take advantage of the ester linkage present in all macrolides and use a water molecule to hydrolyze the bond that cyclizes the macrolactone ring. One possible solution would be to create a macrolide which in place of an ester bond would have the far more stable amide bond. This in turn would no longer allow the Ere enzyme to perform its reaction. However, this will very likely represent a challenge from the point of view of synthesis. Even though the *de novo* synthesis of macrolides has been described using chemical means, the protocol has been developed to utilize the ester linkage (Seiple et al., 2016). Furthermore, also polyketide synthases rely on the creation of cyclizing ester linkage (Park et al., 2010). An alternative approach would be to efficiently block the interaction between Ere and macrolide without impacting ribosome binding. For this to take place it would be helpful to obtain information on the structural details of an Ere-macrolide complex. However, even in the absence of this, we can note that when bound to the ribosome, the ester bond and neighboring atoms do not make specific interactions, and there is space to expand on the macrolide scaffold (Bulkley et al., 2010). It is unlikely that some of the viable expansions could also be accommodated when macrolides bind to Eres, given the need for catalysis, thus providing an avenue to engineer selectivity for the ribosome in the design.

It is appropriate to emphasize the breakthrough that both *de novo* synthesis and protein engineering of polyketide synthases represent. Prior to these two recent developments, efforts to modify the macrolide scaffold were extremely challenging. However, we are now approaching the situation that we are largely limited by our imagination. This places structural information for macrolides interactions with both the ribosome and with the proteins responsible for resistance, at the forefront to efficiently and effectively guide the exploration of macrolide chemical space for the development of next-generations antibiotics.

### Adjuvant Development of Macrolide Therapies

In the context of adjuvant development, it would be optimal to identify inhibitors of various clinically relevant mechanisms of macrolide resistance. Analogous to the design of next-generation macrolides, resistance mechanisms to be potentially addressed are: efflux pump action, alteration of the ribosome, and the action of MPHs and Eres. However, not all these mechanisms are amenable to inhibitor development. Specifically, mutations in the ribosome cannot be addressed through inhibitors. Furthermore, not all enzymes are “drugable,” referring to presence of a distinct pocket that can be uniquely targeted by small molecules. In this respect, the homology model of EreA is discouraging as the pocket shown in **Figure 7** might be too shallow. This leaves efflux pumps, Erm methyltransferases and MPHs as potential targets for adjuvant development. For targeting pumps or ribosome alteration, we refer the reader to Van Bambeke and Lee (2006). Here we will discuss efforts to target MPHs for adjuvant development.

Human ePKs are primary targets for the treatment of cancer and have been extensively explored in drug discovery. To this end the pharmaceutical industry has significantly invested in the design and synthesis of large libraries of compounds that target these kinases. Structural resemblance of aminoglycoside and macrolide phosphotransferases to ePKs, specifically in the nucleotide binding region sparked the idea of repurposing ATP competitive kinase inhibitors against these antibiotic kinases. Soon after the structural homology between APHs and ePKs was uncovered, several known ATP competitive inhibitors of ePKs were assayed for their activity toward APHs. Isoquinoline sulfonamide derivatives, notably CKI-7 were among the first compounds discovered to inhibit some APHs such as APH(3′)-IIIa (Shi et al., 2013). Feasibility of using human protein kinase inhibitors against MPHs and APHs was also tested in a high throughput manner. The screening study clearly showed that although some of the inhibitors can be used against ATP-binding APHs, none were successful against MPHs which are GTP-binding kinases (Shakya et al., 2011).

The observation that an array of protein kinase inhibitors was unable to inhibit MPHs, as these enzymes are GTP-specific, is actually encouraging. A lingering concern in this effort has been the possibility of cross-reactivity of MPH inhibitors with human protein kinases. However, the MPHs’ GTP binding pocket appears to be sufficiently distinct from the ATP binding pocket in ePKs that selectivity is very likely feasible. However, the inability to find leads for MPHs in protein kinase inhibitor libraries implies that leveraging these libraries for adjuvant development is unlikely to be successful. An alternative to a “high-throughput” library screening approaches for inhibitor development is fragment-based drug discovery (Hajduk and Greer, 2007; Lamoree and Hubbard, 2017). This approach has several significant advantages over conventional high-throughput screening campaigns. Most notably, a large segment of chemical space can be surveyed using only a limited number of compounds, and it allows for the discovery of novel scaffolds. In fact, the consensus within the pharmaceutical industry is that fragment-based lead discovery outperforms high-throughput screening approaches as it is more reliable in identifying useful hits and ultimately provides higher quality leads (Hajduk and Greer, 2007). Fragment-based drug discovery does require the three-dimensional structure determination of fragment hits with their target, but this is not an insurmountable obstacle as high-resolution structures of 1.5 Å or better have been obtained for both MPH(2′)-I and MPH(2′)-II (Fong et al., 2017). Given the structural data available for various MPHs, combined with fragment-based screening techniques, it is likely only a matter of time before inhibitors to this class of resistance enzymes will become available. Of course, it is realized that such an achievement would only be the first step in adjuvant development, but nonetheless a critical one in efforts to combat macrolide antibiotic resistance.

## Conclusion

Since their discovery almost 70 years ago, their introduction into clinical practice in 1952 and the first report of clinical resistance 4 years later, much has been learned about macrolides, their mode of action and the various mechanisms by which pathogenic bacteria are increasingly becoming resistant to these antibiotics. This wealth of information now sets the stage for the rational design of much needed therapies that can either overcome or counteract resistance to these antibiotics. The detailed structural insights obtained from the Nobel Prize winning research on how macrolides interact with the bacterial ribosome, combined with the growing information on macrolide resistance mechanisms, enables the rational development of next-generation macrolides. This is furthermore facilitated by recent advances in the synthesis of macrolide variants, either through protein engineering of polyketide synthases, or the *de novo* synthesis. Complementary, detailed structural studies of macrolide resistance mechanisms in conjunction with advanced approaches to inhibitor development, such as fragment-based screening can accelerate the development of macrolide adjuvants. Much research remains to be accomplished, but there is reason for optimism that macrolides may remain a valuable resource to combat bacterial infections.

## Author Contributions

All authors listed have made a substantial, direct and intellectual contribution to the work, and approved it for publication.

## Conflict of Interest Statement

The authors declare that the research was conducted in the absence of any commercial or financial relationships that could be construed as a potential conflict of interest.
